# Amiodarone-Induced Neutropenia: An Uncommon Side Effect of a Common Drug

**DOI:** 10.7759/cureus.10913

**Published:** 2020-10-12

**Authors:** Akshay Kohli, Avinash V Sharma

**Affiliations:** 1 Internal Medicine, MedStar Washington Hospital Center, Washington DC, USA; 2 Cardiology, University of California, San Francisco, Fresno, USA

**Keywords:** amiodarone, neutropenia, agranulocytosis, adverse drug reaction

## Abstract

Amiodarone is widely used as an antiarrhythmic agent for the treatment of both supraventricular and ventricular arrhythmias in various inpatient as well as outpatient settings. Classified as a class III antiarrhythmic agent, it acts mainly by inhibition of potassium channels in the cardiac muscle. Adverse effects are quite common and usually involve pulmonary, gastrointestinal, endocrine, dermatologic, or neuromuscular systems. Although hematologic side effects including thrombocytopenia have also been reported, amiodarone-induced neutropenia is quite rare. We present a case of amiodarone-induced neutropenia in a 66-year-old Caucasian gentleman. He presented to our hospital with cardiac arrest due to ventricular-fibrillation and had received amiodarone as a part of his therapy. His hospital course was complicated by neutropenia which was found to have a clear temporal relation with amiodarone. His initial white blood cell count was 6400/mm3 with an absolute neutrophil count (ANC) of 4800/mm3. His ANC started to downtrend and reached a nadir of 400/mm3 at day six of therapy. This improved significantly after stopping amiodarone, without any change in other medications. Given the rapid improvement of his neutropenia with the discontinuation of amiodarone, further workup with a bone marrow biopsy was not performed. Severe selective neutropenia, also known as agranulocytosis, is a life-threatening condition due to increased risk of severe infections. Antiarrhythmic agents such as tocainamide, procainamide, and flecainide are generally known to cause agranulocytosis. The mechanism of agranulocytosis or neutropenia is thought to be mediated by either immune-mediated destruction or direct and indirect toxicity to myeloid precursors. Although amiodarone has been in use for over 20 years in the management of tachyarrhythmias, agranulocytosis as a direct side effect of amiodarone therapy has been rarely reported. It is important to keep in mind this rare but potentially life-threatening adverse effect of amiodarone when initiating therapy.

## Introduction

Amiodarone is an iodinated benzofuran derivative which is widely used as an antiarrhythmic agent for the treatment of both supra-ventricular and ventricular arrhythmias in various inpatient as well as outpatient settings. Its complex electrophysiologic profile includes prolongation of myocardial repolarization with action on cardiac potassium channels (Class III) as well as class I, II, and IV effects to a lesser degree [1]. While amiodarone is useful in many arrhythmic emergencies and clinical scenarios, its use is sometimes limited due to serious adverse effects. These include pulmonary, endocrinologic, ophthalmologic, neuropathic, and dermatologic effects [2]. Hematologic side effects tend to be rare with even fewer cases of amiodarone-induced neutropenia [3]. Herein, we present a case of isolated neutropenia believed to be induced by amiodarone use.

## Case presentation

A 66-year-old Caucasian man with a past medical history of severe peripheral arterial disease and heart failure with preserved ejection fraction presented to the emergency department (ED) with non-specific complaints of drowsiness and fatigue at work. During the early part of his ED stay, he went into cardiac arrest due to ventricular tachycardia/fibrillation. He was appropriately resuscitated with defibrillation, intravenous (IV) amiodarone, and admitted to the intensive care unit (ICU). His prolonged ICU stay was significant for cardiogenic shock, further tachyarrhythmic events including atrial fibrillation, and post-resuscitation pericardial effusion. He was ultimately diagnosed with ischemic cardiomyopathy from an acute infarction in the territory of his right coronary artery.

On admission, his absolute neutrophil count (ANC) was 6300/mm^3^, this dropped to a nadir of 400/mm^3^ occurring at hospital days five and six. During this time, he was continued on amiodarone for his significant arrhythmic events and transitioned from IV drip to oral (total amount of amiodarone received by the patient was 3450 milligrams). Other medications our patient was on included a proton pump inhibitor, aspirin, atorvastatin, lisinopril, carvedilol, and isosorbide dinitrate. The hematology service was consulted and recommended to discontinue amiodarone as it may be the culprit of his neutropenia. After its discontinuation, his ANC soon started to trend up to 1100/mm^3^, 2000/mm^3^, and 3300/mm^3^ the subsequent three days. Given the rapid reversal of his neutropenia immediately after the discontinuation of amiodarone with no other clinical changes including medication adjustments, this was felt to be the cause of his neutropenia. No further workup including bone marrow biopsy was deemed necessary. Our patient did well and was discharged home.

## Discussion

Neutropenia is defined as an ANC below 1500/mm^3^. Severe selective neutropenia, also known as agranulocytosis, occurs when the ANC drops below 500/mm^3^. This is a life-threatening condition due to increased risk of severe infections with susceptibility to opportunistic microorganisms. Traditionally, neutropenia has been described as transient or acute, and chronic or persistent. Transient neutropenia is usually a result of viral infections, however, drug-induced neutropenia has also been described. Drugs commonly leading to transient neutropenia include, but are not limited to anticonvulsants (carbamazepine, valproate), antimicrobials (sulfonamides, penicillins, trimethoprim/sulfamethoxazole), antipsychotics (clozapine, olanzapine, phenothiazines), and antithyroidals (methimazole, propylthiouracil) [4]. Furthermore, antiarrhythmic agents such as tocainide, procainamide, and flecainide are generally known to cause agranulocytosis [5,6]. Drug-induced agranulocytosis is also characterized into two groups based on the mechanism of agranulocytosis. One that is typically associated with cytotoxic drugs usually manifests in a dose dependent manner, and the other, which is associated with other drugs. The mechanism of agranulocytosis, in this case, is thought to be due to idiosyncratic reaction, either as an immune-mediated reaction or because of direct myeloid cell line damage [7,8]. 

Drug-induced agranulocytosis remains a serious adverse event due to the risk of severe sepsis and septic shock in susceptible populations. Moreover, older age (>65 years), metabolic derangements such as renal failure, and neutrophil count below 100/mm^3^ have been reported to be poor prognostic factors [7]. In our case, although we identified and stopped amiodarone before the neutrophil count went below 100/mm^3^, our patient was at high risk of developing significant complications given his age >65 years and acute renal failure.

Although amiodarone has been in use for more than 30 years in the management of tachyarrhythmias, agranulocytosis as a direct side effect of amiodarone therapy has been rarely reported [3,9,10]. In post-marketing surveillance of amiodarone, the Food and Drug Administration (FDA) mentions this as a potential side effect, however detailed data about this side effect is not available in their report on amiodarone [11].

## Conclusions

Amiodarone is a drug commonly used in clinical practice due to its efficacy in the control of supraventricular and ventricular arrhythmias. It also has adverse effects on several organ systems with hematologic effects including neutropenia, which has been rarely described in the literature. We present a case of such an event where rapid improvement in the ANC occurred upon discontinuation of amiodarone, after being appropriately loaded/dosed. This case serves to bring about further awareness that neutropenia is a potential adverse effect of amiodarone and may warrant prompt discontinuation of the drug. It is very important to have high clinical suspicion for this rare but potentially life-threatening adverse effect when initiating therapy. 
